# Supplementary material for: Are carbon emissions trading and green financial instruments synergistic? -Comprehensive quantitative research based on content analysis
Source: PLoS One. 2024 Mar 7;19(3):e0298601. doi: 10.1371/journal.pone.0298601 (PMC10919601; doi:10.1371/journal.pone.0298601)
Supplement: S1 Data — (ZIP) [file pone.0298601.s001.zip › Data/地级市Z_F工作报告.zip!/╕ú╜¿/╞╬╠∩╩╨/└┤╫╘╣1⁄2╓┌║┼í╛┬φ┐╦╩2╛▌═°í┐_2010.docx]

各位代表：

现在，我代表莆田市人民政府向大会报告工作，请予审议，并请市政协各位委员和其他列席人员提出意见。

一、化危为机 逆势奋进 湄洲湾港口城市发展呈现新气象

2009年，在中共福建省委、省人民政府和中共莆田市委的坚强领导下，我市各级政府深入学习实践科学发展观，认真贯彻落实党中央、国务院和省委、省政府关于建设海峡西岸经济区的战略部署，以及刺激经济增长的一系列政策措施，按照“四用发展”和“三个持续”要求，大力实施“以港兴市、工业强市”发展战略，着力保增长、保民生、保稳定，统筹协调，整体推进，迎难而上，共克时艰，有效化解全球金融危机的不利影响，较好地完成了市五届人大四次会议确定的各项目标任务。

（一）经济持续较快增长，综合实力稳步提升。初步核算，全市生产总值691.7亿元，增长14.5%；全社会固定资产投资362.7亿元，增长20.2 %；财政总收入63.2亿元，增长19.7%，其中地方级财政收入37.9亿元，增长28.3%；规模工业产值959.3亿元，增长20.7%；社会消费品零售总额240.6亿元，增长15.8%；城镇居民人均可支配收入17308元，实际增长11%；农民人均纯收入6921元，实际增长8.6%；居民消费价格指数99.1%，低于年初预期3.9个百分点；城镇登记失业率2.5%；人口自然增长率6.7‰。经济结构继续优化，三产比例调整为11.1：57.5：31.4。农林牧渔业总产值128亿元，增长5.4%。农业产业化水平进一步提升，120家市级以上龙头企业带动农户50万户，实现产值130亿元。规模工业企业净增168家，总数达1166家。轻重工业结构进一步优化，重工业比重提高2.9个百分点。第三产业增加值216.9亿元，增长15.4％。金融、商贸、房地产、滨海旅游等产业较快发展。年末全市金融机构本外币存款余额608.8亿元、贷款余额497.3亿元，分别增长25.6%、42.5%。新增中小企业融资担保机构10家。发放“家电下乡”补贴1897万元，带动家电消费1.5亿元。鞋服城等专业市场顺利开业，沃尔玛等大型超市正式营业。中国（莆田）工艺美术城被评为国家4A级旅游景区。项目带动战略成效显著，285个在建、预备重点项目完成年度投资228.6亿元。争取中央增投项目158个、资金3.3亿元，安排地方政府债券配套资金2.2亿元。湄洲湾港口加快开发，11个深水泊位开工建设，5万吨级木材专用码头竣工投产。向莆铁路莆田段、湄洲湾港口铁路支线、莆田燃气电厂、石城风电场、石井风电场、南日风电场、仙游抽水蓄能电站、金钟水利枢纽工程、木兰溪防洪工程二期霞林段、市污水处理厂二期扩建等重大项目加快推进。国投湄洲湾（石门澳）产业园、莆永高速莆田段、城区大型平战结合人防工程、闽中食品工业园顺利开工。新增中国驰名商标1个、国家出口免验产品1个、省名牌产品16个、省著名商标20个，制订国家标准6项、行业标准3项、福建省地方标准10项。度尾文旦柚获得国家地理标志产品。中国鞋业创新示范基地落户莆田。我市再次被中国城市品牌大会评选为“中国最具发展潜力城市”。

（二）城乡建设步伐加快，市容村貌明显改观。城镇化进程加快，城镇化水平达47.2％，提高0.9个百分点。实施城建项目94个，完成年度投资85.7亿元。完成新一轮土地利用总体规划、城市总体规划，修编城市“绿心”等专业规划。城市“三纵四横”主干道路、19条县（区）市政道路建设加快，福厦铁路莆田段、莆秀高速公路、城港大道一期、迎宾大道、莆兴路等13条道路竣工通车。创建国家园林城市工作扎实推进，一批供水、供气、园林、夜景、路灯等公共设施加快建设。护城河五期等片区加快改造，安置房回迁4125户。省运会场馆和星级酒店等配套设施基本建成，城市品位显著提高，城区面貌焕然一新。大力治理出租车市场，新增一批公共交通设施，市民出行更加安全方便。惠农政策全面落实，市级以上财政投入“三农”资金15.2亿元，增加3.2亿元，增长26.7％，减轻农民负担2.3亿元。加快新农村建设，累计完成38个集镇、600个中心村总体规划和332个村镇住宅片区详细规划。继续建设100个新农村示范村，着力帮扶20个重点贫困村。782个行政村实施农村家园清洁行动，农村生活垃圾无害化处理率达80%以上，被省政府评为“农村家园清洁行动先进市”。农业综合生产能力增强，建设高标准农田3万亩，新增节水灌溉2万亩，治理水土流失3.7万亩，除险加固水库24座，强化加固海堤20.6公里，整治城乡河道57公里，新建扩建标准渔港3个。建成农村水泥路399公里、农村户用沼气池7570口。继续实施农村饮水安全村村通工程，惠及人口90.5万人。“造福工程”、“阳光工程”分别完成省下达3700人、7200人任务。落实大型库区移民后期扶持资金3816万元。镇海街道、长寿社区和常太镇岭下村分别获“全国和谐社区建设示范街道”、“全国和谐社区建设示范社区”和“全国巾帼示范村”称号。

（三）改革开放继续深化，发展活力不断增强。土地承包经营权、集体林权、海域使用权等农村综合改革全面推开。完成522个行政村的集体林权改革任务，发证面积299万亩，发放公益林补偿资金832万元。“三百工程”顺利开展，农村“六大员”、党员干部下派驻村、村干部保障制度不断完善，村干部养老保险制度逐步推行，村主干每月最低工资不少于600元。国有资产监管体系逐步完善，国企改制工作稳步推进。投融资体制和税费等重点领域改革不断深化，组建一批交通、体育等行业的市级投融资公司和国有产业集团。农村信用社顺利改制，莆田农村商业银行加快筹建。公用事业市场化行为不断规范，户外广告清理工作成效明显。公共财政体系建设得到加强，政府采购改革取得实效。22家企业上市工作有序推进，3家公司具备境外上市条件，7家公司完成股份制改造。湄洲湾港口一体化有效运作。莆田台商投资区前期工作进展顺利。仙游台湾农民创业园升格为国家级台湾农民创业园。对外经贸平稳发展，招商项目推介会、艺博会、木洽会、鞋交会、东南亚经贸活动周、海交会、商交会、投洽会等经贸活动成果丰硕，实际利用外资6.6亿美元（历史可比口径），增长17.1%；外贸进出口总额23.5亿美元，增长1.1%，其中出口总额16.7亿美元，下降2.4%，降幅小于全省平均水平4个百分点。成功举办妈祖文化旅游节、南少林武术文化节、海峡论坛——台湾妈祖信众直航湄洲进香活动。湄洲岛与嘉义布袋港首次客运直航，秀屿港与基隆港首次货运直航。莆港澳合作纵深拓展。对口支援和支持彭州濛阳镇灾后重建工作取得新成效。

（四）节能减排扎实推进，生态环境逐步优化。节能减排投入13.3亿元，增长5.5%。持续推进重点行业、企业和领域节能减排，严格控制高耗能、高污染项目，淘汰一批落后产能。市控20家重点企业能耗下降3%，节约3.5万吨标准煤。工业用水重复利用率、固体废弃物利用率、清洁能源结构比重分别提高到65%、70%、49%。实行最严格的耕地保护制度，继续清理工业闲置土地，拆除违法占地建筑5.5万平方米，审批建设用地16951亩，开发整理土地2.2万亩，连续10年实现耕地占补平衡。环保基础设施进一步完善，湄洲湾电厂等3个脱硫项目和仙游城区污水处理厂投入运行，4座污水处理厂加快建设，新铺设污水管道172公里，新增城市污水日处理能力11万吨。市垃圾焚烧发电厂、大湖垃圾处理场扩建工程加快推进，仙游寨岭垃圾无害化处理场投入使用。农业面源污染有效治理，东圳、外度库区等重点水域的畜禽养殖污染整治得到加强，全市拆除禁养区内畜禽场857家、面积41万平方米。植树造林9.5万亩，森林覆盖率达57.3%。城市空气质量达到二级标准。海洋水环境质量保持全省最好水平。单位生产总值能耗下降3％。二氧化硫排放量下降8.37％，化学需氧量排放减少0.51％，超额完成省下达主要污染物年度减排计划。

（五）社会事业全面进步，人民生活有效改善。市级以上财政投入民生社会事业52.9亿元，增长42.9%。11件为民办实事项目基本完成。科技创新能力增强，第三次蝉联“全国科技进步先进城市”称号。取得授权专利225项，“菲律宾蛤仔现代养殖产业技术体系的构建与应用”获得国家科技进步二等奖。中国鞋业信息中心、中国鞋类设计与研发中心落户莆田。素质教育全面推进，教育质量进一步提高。落实义务教育经费12.5亿元，化解农村义务教育债务2.9亿元，免除义务教育公办学校学生学杂费1.3亿元，公办义务教育阶段享受免费教科书学生比例提高到87.2%，受益32万人。农村留守、流动儿童和校园绿色网室工作成效明显。实施中小学校舍安全工程13万平方米。城乡公共卫生、医疗服务和药品供应保障体系更加完善，社区卫生服务中心覆盖率达100%。参加新型农村合作医疗245万人，参合率达96.5%。免费接种甲型H1N1流感疫苗12万人，甲流疫情得到有效防控。提升改造乡镇卫生院12家。计划生育优惠政策得到落实，继续保持低生育水平，创建计划生育优质服务先进县（区）和后进转化工作得到加强，城厢区顺利通过全国计划生育优质服务区考评。市档案馆晋升为国家一级档案馆。完成非物质文化遗产普查工作。“妈祖信俗”成功申报世遗。莆仙戏创作表演取得新成就，《春草闯堂》获文化部“优秀保留剧目”奖。中央电视台中秋晚会在湄洲岛成功举办。新闻出版、广播影视、社会科学取得新进展。第十四届省运会有序筹备，全民健身活动广泛开展。群众性精神文明创建活动深入开展，获得“省第十届创建文明城市工作先进城市”称号。实行积极的就业政策，新增城镇就业2.2万人，转移农村劳动力3.3万人。城乡社会保障体系不断完善，企业基本养老、城镇职工和居民基本医疗、失业保险参保人数分别达20.1万人、62万人和15.3万人。城镇居民和职工医疗保险扩大补助范围，提高补助标准。发放城乡低保金1.05亿元。向9462户城乡低保户实施医疗救助，发放医疗救助金620万元。新建农村敬老院6所。建设廉租住房6.8万平方米，发放廉租住房租赁补贴142万元，惠及1300户城市低收入家庭。完成侨居造福工程5.3万平方米。加大“平安莆田”建设力度，完善社会治安防控体系，严厉打击严重暴力犯罪、“两抢一盗”等违法犯罪活动。高度重视安全生产、信访和多元化矛盾纠纷调解衔接工作，维护国家安全和社会稳定。巩固“全国双拥模范城”三连冠的共建成果，军政军民更加团结。国防动员、民兵、预备役、海防等工作持续推进。人事、民族、宗教、保密、统计、气象、水文、地震、科普、地方志等工作稳步发展。

过去的一年，我们进一步加强政府自身建设。基层民主进一步健全，村（居）委会和社区换届选举顺利进行，村（居）换届选举完成率97.7%，社区换届选举完成率100%。“五五”普法深入开展，依法行政加快推进。认真贯彻市人大及其常委会的各项决议，认真办理人大代表的建议、意见和政协提案，办结率均为100%。政务公开和政府信息公开工作扎实推进。强化应急体系建设，有效应对突发公共事件。组建新的市行政服务中心，建立网上审批和电子监察系统，开展第八轮清理行政审批项目工作，削减许可事项110项，压缩审批环节179个，平均每个事项压缩7.8个工作日。全面推行领导任期经济责任审计制度，查纠违纪金额2619万元。深化机关效能建设和绩效管理，纠正损害群众利益的不正之风，加强反腐倡廉工作。

各位代表！过去一年，在加快建设湄洲湾港口城市的进程中，我们取得了新成效。这是省委、省政府和市委正确领导的结果，是全市人民和衷共济、奋力拼搏的结果。在此，我代表市人民政府，向全市广大干部群众，向人大代表、政协委员和离退休老同志，向各民主党派、工商联、各人民团体、无党派人士和社会各界人士，向中央及省驻莆单位，向驻莆部队指战员、武警官兵和公安干警，向所有支持和关心莆田发展的港澳台同胞和海内外朋友，表示崇高的敬意和衷心的感谢！

在肯定成绩的同时，我们也清醒认识到发展还面临不少困难和问题，主要是：我市经济总量仍然偏小，发展速度不够快，受宏观经济环境影响，部分主要经济指标增速回落；产业规模不大，支柱产业不强，新兴产业项目不多；自主创新能力不强，现代服务业发展滞后，产业结构调整优化更加紧迫；工业化、城镇化水平仍然不高，以工促农、以城带乡能力不强，统筹协调发展的任务还很艰巨；对外开放整体水平不高，国际市场需求低迷态势短期难以改变，外贸出口和吸引外资困难加大；城乡规划执行仍需加强，违法违规建设问题依然存在，城市建设和管理水平有待提升；重点流域水环境综合整治、重要饮用水源地环境保护工作仍然艰巨，垃圾、污水处理项目进展较慢，节能减排压力加大；农业基础仍不稳固，农民持续增收难度较大；社会事业投入不足，就业结构性矛盾突出，民生保障水平不高；信访问题比较突出，社会安定稳定存在不确定因素，安全生产隐患犹存；政府自身建设仍然存在一些问题，干部作风和办事效率不尽如人意，个别领域还存在腐败现象。对此，我们忧患在心，将采取有力措施认真解决，绝不辜负全市人民的期望和重托。

二、融入海西 乘势而上 加快推进湄洲湾港口城市建设

2010年是实施“十一五”规划的最后一年，也是我市深入贯彻党中央、国务院和省委、省政府加快建设海峡西岸经济区战略部署的关键一年。做好今年工作，保持经济社会平稳较快发展，加快推进湄洲湾港口城市建设意义重大。今年政府工作的总体要求是：全面贯彻党的十七大、十七届三中、四中全会、中央经济工作会议和省委八届七次全会、全省经济工作会议精神，以邓小平理论和“三个代表”重要思想为指导，深入贯彻落实科学发展观，按照“五个更加注重”的要求，站位海西、融入海西，全面实施“以港兴市、工业强市”发展战略，紧紧抓住经济工作中的重点、难点和薄弱点，好中求快，又好又快，有效转变，全力突破，持续提升，在转变发展方式、优化经济结构、强化项目带动、建设宜居城市、扩大对外开放等方面取得新突破，加快推进湄洲湾港口城市建设，为海峡西岸经济区建设作出新的贡献。

经济社会发展的主要预期目标是：努力实现“保八超十，全面实现‘十一五’规划目标”，全市生产总值确保实现800亿元以上，财政总收入增量超过10亿元，“十一五”规划确定的目标任务全面完成。全市生产总值增长15%；规模工业产值增长20%；全社会固定资产投资增长29.5%；财政总收入增长16%，其中地方级收入增长11%；社会消费品零售总额增长16%；外贸进出口总额增长6.5%；实际利用外资（历史可比口径）增长10%；城镇登记失业率控制在3%以内；城镇居民人均可支配收入和农民人均纯收入分别增长8%左右；居民消费价格指数涨幅控制在3%左右；人口自然增长率控制在7.5‰以内；实现生产总值能耗降低3%；完成二氧化硫和化学需氧量年度减排任务。

今年要着力抓好以下八项工作：

（一）统筹城乡发展，推进城乡一体化进程

加快城乡建设。坚持把“三农”工作放在重中之重的位置，贯彻党的农村基本政策，夯实农业农村发展基础，协调推进工业化、城镇化和农业现代化，统筹城乡空间布局、产业发展、基础设施、社会事业和环境建设，努力形成城乡经济社会发展一体化新格局。加快农村路网建设，改造农村普通公路300公里。建成木兰溪防洪工程二期霞林段，加快建设金钟水利枢纽引水配套工程和木兰溪防洪工程三期荔涵段。深化乌溪水库前期工作。续建大中型灌区配套和节水改造工程，除险加固病险水库21座、海堤22公里。新建平海一级渔港等标准渔港。发展农村户用沼气5680口。巩固新农村建设成果，抓紧编制村镇规划，统筹安排村庄建设用地和宅基地，清理违法建设和违法占地建房，引导农民向城镇集聚，预留临港工业和城市发展空间。健全农村教育、卫生、文化等设施，发展农村社会事业。加强农村信息化建设。

发展现代农业。转变农业发展方式，调整优化农业结构。稳定粮食播种面积80万亩。加快实施新产品、新技术、新肥料、新农药、新机具“五新”推广示范工程，为农民提供全方位的技术服务。提升六大优势产业，大力发展水果、水产、畜牧、食用菌、蔬菜、林业等特色农产品。结合农村生态环境保护，建设一批生猪、蛋鸡、鱼虾等标准化、规模化的健康养殖示范场。创建农产品加工示范园区，扶持产业化龙头企业，提高农产品精深加工水平，促进加工流通增值。实施农业标准化，发展品牌农业，提高市场营销水平。推行无公害农产品、绿色食品、有机食品等农产品标识认证制度，积极实施国家地理标志产品保护。扶持发展仙游台湾农民创业园，加强莆台农副产品交流合作。

落实惠农政策。按照总量持续增加、比例稳步提高的要求，不断增加“三农”投入。继续落实农业政策，保障各类补贴资金及时足额到位。积极抓好被征地农民的就业培训和社会保障等工作，确保失地农民安居乐业。继续做好20个重点村的扶贫帮困工作。实施“造福工程”，建设游洋镇石里村、萩芦镇潭井村等省造福工程示范点。创新农村工作机制，完善干部驻村任职、农村“六大员”等制度，提高村主干待遇。

（二）实施项目带动，争创持续发展优势

突破重大项目。着力提高工业项目、产业化项目在固定资产投资中的比重，认真抓好302个在建、预备和前期重点项目，确保完成年度投资计划。提高项目策划生成水平，加大项目推介力度，用足用够中央、国家部委和央企支持海西建设政策措施，紧密对接世界500强企业，力争引进一批投资几十亿、上百亿的重大项目。专人专职负责，有效有力协调，强力推进鞍钢冷轧、林浆纸一体化等确保开工和争取开工的“双十”项目，支撑持续增长，引领产业集聚，带动区域发展。

加快港口建设。续建新建东吴、秀屿、罗屿、莆头作业区等17个深水泊位和三江口3000吨级码头。加快国投湄洲湾（石门澳）产业园基础设施工程、国投湄洲湾煤炭码头一期工程、罗屿30万吨级干散货码头建设。扩建湄洲岛对台客运码头，改造忠湄轮渡码头和候船大楼。建成湄洲湾航道二期工程，新建三江口3000吨级航道。认真做好新一轮海洋功能区划调整工作，完成兴化湾南岸港口和产业布局规划。依法清理闲置临港土地和岸线，集约利用港口资源。

构建交通网络。建成莆田铁路枢纽站。续建向莆铁路莆田段、湄洲湾港口铁路支线。深化福厦铁路货运专线、福厦城市轻轨莆田段、兴化湾南岸港口铁路支线前期工作，以及国投配煤基地、秀屿国家级木材贸易加工示范区和仙游工业园区3条铁路专用线前期工作。扩建沈海高速公路莆田段，续建莆永高速公路莆田段，开工建设沈海高速复线莆田段和兴尤高速公路莆田段。按照“储万亩地，融百亿资，建疏港路”的目标，加快建设城港二期、涵港、仙港、沁峤、201省道、202省道等24条疏港道路，尽快形成连接中心城市与港口、工业开发区、生态旅游度假区的快速通道。积极筹建莆田机场。

建设能源基地。加快推进LNG汽车及船舶加气站、莆田燃气电厂、仙游抽水蓄能电站、南日风电场三期、石城风电场二期、石井风电场二期、东峤风电场、35千伏输变电等项目。开工建设忠门风电场、南日风电场四期等项目。扎实做好莆田核电、海上风电、湄洲湾电厂二期、东吴港区成品油储备基地等项目前期工作。编制并实施电力走廊规划。促进能源产业转型升级，延长产业链，形成多品种的能源结构，构建海峡西岸经济区的能源供应、石油储备和煤炭中转基地。

（三）调整优化结构，促进产业转型升级

培育先进制造业。完善工业发展考核办法，加大工业项目推进力度，增强工业项目在产业发展中的支撑作用。认真对接国家、省产业调整和振兴实施方案，完善湄洲湾（石门澳）产业园和LNG、LCD、LED等产业园规划，改造提升鞋服、食品、电子、工艺美术等传统优势产业，扶持发展冷轧钢板、石化、光电、新材料、新能源和精密机械、大型装备制造等新兴产业。跟踪落实40个新增产值5000万元以上的省级新增长点项目，新增产值30亿元。推进炼化一体化、冶炼、修造船、林浆纸、大型磷钾肥等重大临港工业项目前期工作。争取国家和省在湄洲湾北岸、兴化湾南岸布局大型重化工业项目和机械制造项目。

做大做强企业。鼓励企业通过上下游对接、改制上市、同业联合、重组兼并等方式，扩大规模，壮大实力。积极保护和创建区域品牌和产品品牌，新增国家级品牌2-3个、省级品牌40个以上。建立本地名优地产品推荐目录，加大对本地名优地产品购销力度。鼓励企业运用电子商务平台，大力拓展市场。规范行政事业性收费，减少涉企收费，减轻企业负担。用好工业发展专项资金，扶持快速成长型企业，培育一批产值超20亿元以上企业，新增规模以上企业100家以上，力争总数达到1300家。

提升现代服务业。落实现代服务业发展政策，促进服务业拓宽领域、优化结构、提速发展、做大总量。围绕打造东南沿海重要物流节点城市目标，加快建设一批临港工贸物流园、物流配送中心和大型专业市场，继续完善工艺美术城、鞋服城、涵江国际商贸城等平台，突出发展现代物流业。培育发展软件和信息服务业，促进物联网产业发展，扩大服务业的对外开放，发展服务外包。扶持发展会计、审计、律师、公证、评估、代理、经纪等各类中介服务业。制定优惠政策，吸引境内外企业在莆田设立地区总部、销售中心、结算中心和研发中心。大力发展批发零售、住宿餐饮等传统行业。积极发展医疗保健、家政服务、物业管理、社区服务等服务业，培育文化娱乐、体育健身、技术培训等新消费热点。落实促进房地产市场健康发展的政策，规范房地产市场秩序，扩大多层次的住房消费。继续实施万村千乡市场工程，推进家电、汽车、摩托车下乡，促进农村消费。

拓展旅游经济。围绕建设海峡西岸滨海文化旅游度假胜地目标，加大旅游资源开发力度，创建湄洲岛国家5A级旅游景区，加快南少林、九鲤湖、九龙谷、南日生态旅游岛、白沙温泉度假村等项目建设，提高旅游综合配套能力。打响旅游品牌，策划精品线路，加强旅游合作，开拓旅游市场，发展特色旅游。大力发展森林之家、农家乐、渔家乐等乡村生态休闲旅游。

推进自主创新。创建一批国家重点实验室、国家质检中心、产业技术（研发）中心，建成国家浆纸质检中心一期工程，开工建设国家浆纸质检中心二期、省木雕古典家具质检中心。鼓励企业建立技术中心、研究中心、产品检验检测中心，参与制修订国家、行业和省地方标准。创建10家市级以上企业技术中心，滚动实施30个市级企业技术创新重点项目。积极利用“6·18”平台，举办工艺美术项目等对接会。继续建设创新型城市，提升院地合作水平，推动产学研联合，提高企业自主创新能力。支持新兴产业、高新技术产业建立博士后站点，鼓励高层次人才、出国留学人员来莆创业，培养引进优秀科技领军人才。

（四）推进改革开放，增强持续发展活力

提高招商质量和水平。以台港澳和东南亚等地区为重点，突出产业链招商，引导外资投向先进制造业、高新技术产业、现代服务业、新能源和节能环保产业。创新利用外资方式，引进境外投资基金，支持外资并购。跟踪落实36个在谈千万美元以上项目，实现利用外资新突破。办好艺博会、木洽会、鞋交会、妈祖文化旅游节等经贸活动，积极参与海交会、商交会、投洽会等知名展会。充分发挥各地莆田商会和莆籍民营企业家作用，推动民资回归。

保持进出口稳定增长。积极运用出口退税、出口信用保险等外经贸扶持政策，增强外贸企业竞争力，促进加工贸易企业转型升级。支持企业开展国际认证注册，加入跨国采购链，做大传统市场份额，培育新兴市场，开拓东盟自由贸易区市场。鼓励企业进口重要资源、先进技术和关键设备。积极建设鞋革、木材、电子、天然气、大豆等国家级进出口产业基地。力争扩大莆田一类口岸开放范围，优化口岸通关环境。支持有条件的企业“走出去”，开发利用境外资源和市场。

推动开发区创新发展。继续提升湄洲湾北岸经济技术开发区、高新技术产业园区、仙游经济开发区以及各工业集中区建设水平，规划建设现代商务集聚区，加快功能开发，重视环境保护，提高配套能力，促进产业集聚。争取设立海关特殊监管区，积极创建台商投资区、出口加工区和保税物流园区。

加强对外交流合作。用足用好先行先试政策，发展对台小额贸易，培育莆台商贸流通企业，促进优质地产品双向流通。积极参与海峡论坛，办好两岸妈祖文化活动周。增进莆台政党、行业协会以及科技、文化、教育等领域的交流合作。创建妈祖文化产业园。争取台湾妈祖进香团直航湄洲岛自由进出免签注，发展“金马澎”旅游。办好“香港招商周”活动，加强与港澳投资贸易合作，联手拓展国际市场。发挥侨乡优势，密切联系海外华侨华人。组建市政府驻南昌办事处、长沙办事处，加强与向莆铁路沿线城市合作，探索建立更加密切的合作机制，促进资源优化配置。积极参与闽浙赣皖经济协作区、闽东北协作区交流活动。

深化重点领域改革。编好“十二五”规划。完善政府投融资体制，继续抓好规划、评估、审核、交易等制度建设。继续深化供水、农资、医疗卫生体制改革和重要商品价格改革，抓好市区自来水企业股份制改革，提高企业的经济社会效益。完善国有资产管理体制和投资决策、风险控制和责任追究制度，依法履行出资人职责，保障出资人权益。深化政府项目代建制和政府采购改革。支持企业改制上市和发行债券。加快农村综合改革，重点抓好江口镇、西天尾镇2个省定小城镇综合改革建设试点, 鼓励各县区选择1-2个乡镇作为小城镇综合改革建设试点，加快城镇化进程。

（五）创新财经工作，提高科学理财水平

稳定财税收入增长。大力引进税收贡献率高的重大工业项目，着力扶持支柱产业，培育发展大型企业集团，加快发展房地产、金融等服务业，发展税源型经济。着力抓好餐饮、娱乐、私房租赁等行业的税收征管。强化税收属地征管，依法治税，抓好大宗税源、重点税种征收工作。建立财、税、库、银税收收入电子缴库联网系统，防范和打击偷逃税行为,确保税款及时足额入库。盘活城市存量土地，加大土地收储力度，清缴土地出让金，增加政府性基金收入。

提高公共财政绩效。建立公共财政和预算绩效评价体系，研究制定市级财政支出绩效评价办法，提高资金使用效益。加大对困难县（区）转移支付力度。优化财政支出结构，厉行勤俭节约，严格控制公务购车、会议、公务接待、党政机关出国(境)经费支出，把更多的资金用到促增长、调结构、惠民生上来。加强政府债务管理，防范债务风险。

推进金融机制创新。鼓励金融机构创新金融产品和服务，有效增加信贷投放，加大对产业升级、中小企业、节能减排、三农、外贸出口等重点领域的信贷支持力度。创新信贷产品和经营模式，积极推广银团贷款、联合贷款、委托贷款，支持重点项目建设。组建莆田农村商业银行。鼓励金融机构来莆田设点，支持银行业扩张网点，完善银行业服务体系。引导邮储银行扩大小额贷款试点，促进邮储资金回流农村。支持开展以动产、权利为抵质押的信贷业务。新成立10家担保公司。创新保险公司融资担保模式，开展中小企业贷款履约保证保险工作。

（六）建设环保家园，打造滨海宜居城市

完善城市功能。保护壶山兰水景观，突出荔林水乡特色，建设生态环保家园，打造滨海宜居城市。编制滨海宜居城市建设规划以及荔城、涵江新区规划，实施城市生态绿心保护利用规划，开展木兰溪、迎宾大道、城港大道两侧等城市设计，制定城市色彩、夜景、绿化景观廊道规划和建筑风格导则。规划建设木兰溪滨江新城、盘龙山庄、萩芦溪山庄、樟林水乡、白塘湖和土海综合开发等项目，完成木兰溪下游宁海挡潮闸项目论证工作，启动木兰溪北洋人工湖项目前期工作，打造亲水生态城市。加快改造“城中村”步伐，实施南郊濠浦等12个片区旧城改造工程。继续开展市政整治工程，重点建设一批城市道路、供水、供气、园林、夜景、路灯、公交、停车场、污水垃圾处理工程，新增市政道路30公里、日供水能力8万吨、燃气管道45公里、园林绿地101公顷、路灯12公里，铺设污水管道276公里，污水日处理能力达到22万吨，垃圾无害化日处理能力达到850吨。继续推进妈祖城建设。

落实节能减排责任。重点抓好燃煤工业锅炉改造、电机改造、建筑节能、绿色照明、政府节能等十大领域60项节能重点工程，推进10家省级循环经济示范企业、2个循环经济示范园区、9家清洁生产试点单位节能减排工作。力争工业固体废物综合利用率达75%以上，工业用水重复利用率达70%以上。着力整治造纸、石材、重金属、涉铅、制革、电镀、印染、食品、制药、化工等行业的主要污染物，促进企业达标排放和持续减排。大力推进全国节水型城市试点建设。

加强生态建设和保护。实施“绿色屏障、绿色通道、绿色园区”生态工程，优化森林资源结构，提升森林资源生态功能。建设木兰溪源头、老鹰尖自然保护区。抓好东圳、外度、金钟水库等饮用水源保护区人口搬迁、清污截污、退耕还林工作，确保饮水安全。合理开发利用矿产资源，治理水土流失，严厉打击非法采矿行为。大力实施土地整理增减挂钩和耕地开发项目，实现耕地总量动态平衡。保护海洋生态环境，持续发展海洋经济。继续建设湄洲岛生态文明示范基地。

强化环境综合整治。继续推进木兰溪、萩芦溪流域水环境综合整治，控制畜禽养殖污染。建成市垃圾焚烧发电厂、医疗废弃物处理厂以及闽中污水处理厂二期、枫亭、荔城、秀屿、北岸、湄洲岛污水处理厂。加快污水管网建设，强化污水排放收集管理，大力整治南北洋河道，净化城区内河水质。继续实施餐饮业油烟、汽车尾气、建筑施工、噪声污染等专项整治，改善环境质量。深入开展846个村（居）农村家园清洁行动。推广生态农业生产模式，治理农村面源污染。

（七）繁荣社会事业，构建和谐文明社会

办好省运会。今年我市将承办第十四届省运动会，这是省委、省政府和全省人民的信任与重托。要做好各项筹办工作，加强城市建设管理和精神文明建设，改变城乡面貌，提高人民素质，提升群众体育和竞技体育水平。努力建设一流的场馆设施、开展一流的赛事组织、营造一流的环境氛围、提供一流的服务保障、争创一流的比赛成绩，办好一届有特色、上水平的省运会。

优先发展教育事业。促进义务教育均衡发展，全面达到“双高普九”要求。加快城乡义务教育学校标准化建设，落实义务教育教师校际交流和城镇教师农村任教服务制度。续建中小学校舍安全工程，改造校舍192万平方米。迁建仙游师范学校、市电大和莆田职校。大力发展职业教育，培养技能型紧缺人才。提升高等教育办学水平，加强重点学科建设。重视发展学前教育和特殊教育，规范民办教育，加强终身教育和社区教育。

提高人民健康水平。加强公共卫生服务体系建设，完善农村三级医疗卫生服务网络和城市社区卫生服务网络，提高公共卫生服务能力。全面推行城镇居民、城镇职工基本医疗保险，巩固完善新型农村合作医疗制度，健全医疗救助体系。强化重大疾病防治和卫生应急管理，做好甲型H1N1流感防治工作。加快建设莆田学院附属医院新区、市第一医院门诊大楼、市妇幼保健院，改建扩建3所县区级医院，新建改造一批镇卫生院和村卫生所。提高医疗质量和服务水平，构建和谐医患关系。建设“食品放心工程”，严格食品、药品监管，让人民群众吃得安心，用得放心。认真开展第六次全国人口普查。统筹解决人口问题，不断加大创建计划生育优质服务先进县（区）和后进转化工作力度，稳定计划生育政策和低生育水平，提高出生人口素质。

加快发展文化事业。实施文化惠民工程，续建莆仙大剧院、博物馆、市艺校，改造43个乡镇文化站。认真办好中央电视台“心连心”艺术团赴湄洲岛慰问演出活动。做好第三次全国文物普查和“妈祖信俗”等非物质文化遗产保护工作。组建莆仙戏剧院。培育发展文化创意产业，打造莆田特色文化品牌。繁荣发展哲学社会科学、新闻出版、广播影视、文学艺术事业。

保持社会和谐稳定。持续建设“平安莆田”，加强社会治安综合治理，着力抓好社会矛盾化解、社会管理创新、公正廉洁执法三项重点工作，强化对重点人口、重点物品、重点阵地的管理。完善打防控机制，加快城乡社区警务建设，依法防范和打击各类违法犯罪活动。加强国家安全工作，防范和严厉打击境内外敌对势力渗透破坏活动。继续开展“五五”普法、法律服务和法律援助。进一步加强保密工作，全力维护国家安全和利益。健全重大项目安定稳定风险评估等维护群众权益机制。深化信访“五抓”机制，推动信访工作步入法制化、规范化管理轨道。强化安全生产“一岗双责”和企业主体责任，防止重特大事故发生。完善突发公共事件应急处置机制和预案体系，提高防灾减灾和处置突发事件的能力，保障公共安全。支持边防部门实施“爱民固边”战略，创建和谐安全的边防环境。加强海防、人防工作和民兵、预备役建设，妥善安置复员转业军人，支持驻莆部队和武警部队加强训练，提高“双拥共建”水平。重视对口援建，确保完成任务。支持工会、共青团、妇联等人民团体依照法律和各自章程开展工作。认真落实党的民族政策，依法管理宗教事务。积极发展人事、档案、统计、气象、水文、地震、科普、老龄、残疾人、红十字、地方志等各项社会事业。

（八）强化民生保障，共享改革发展成果

稳定和扩大就业。统筹城乡就业，继续落实促进就业政策，支持自主创业，以创业带动就业。开辟公益性岗位，提供更多就业机会。加强对城乡零就业家庭的就业援助，帮助失业困难群众再就业。做好大中专毕业生就业工作，重点帮助特困家庭毕业生就业，引导和鼓励毕业生面向基层就业。完善就业服务体系，继续做好跨区域劳务合作。实施就业培训工程，新增城镇就业2.1万人，转移农村劳动力3万人。

保障城乡群众生活。提高企业退休人员养老金待遇。积极推进新型农村社会养老保险试点工作。加强社会保障能力建设，逐步实现社会保障卡在医疗等领域一卡通用。扩大城镇职工养老、医疗和失业保险覆盖面。完善城镇居民基本医疗保险政策，年内覆盖率达95%以上。落实农民工养老保险跨区域转移工作。建成市慈康医院、市社会福利中心。新建扩建4所敬老院。加大保障性住房建设力度, 筹建廉租住房1390套，解决2190户低收入家庭居住困难，回迁安置房2669户。

办好为民实事。集中力量办好中小学校舍安全、饮水安全村村通、创建优美人居环境、残疾人康复扶贫助学安居、基本医疗服务和公共卫生服务体系、多元化调解衔接工作机制和百个农村法律服务基点户、整顿交通秩序、10千伏配网、无线城市、电信网络升级改造等10件关系人民群众切身利益的实事。

三、干事创业 执政为民 努力建设人民满意的政府

各位代表，我们的政府是人民的政府，实现最广大人民群众的根本利益是各级政府的最高责任和追求。我们一定要殚精竭虑，勤奋工作，永不懈怠，用为民、务实、清廉的实际行动，努力建设为人民服务、对人民负责、让人民满意的政府。

奋发有为，提高科学发展的执行力。加强学习，努力建设学习型政府。坚持科学发展的理念，始终保持时不我待的干劲、敢为人先的闯劲、一抓到底的韧劲，把干事创业的热情与科学求实的态度结合起来，推动经济社会又好又快发展。坚持一切从实际出发，深入一线察实情，沉下身子出实招，开动脑筋破难题，努力创造出实实在在的业绩。坚持一切按规律办事，不断提高对市场经济运行、现代产业发展、新型社会管理等规律的认识，遵循发展规律，理清发展思路，创新发展举措，创造出经得起实践检验的政绩。坚持一切依靠群众、一切为了群众，以民心为己心，以民需为己求，以民生为己任，努力创造让群众满意的业绩。

依法行政，提高促进发展的保障力。始终把依法行政贯穿于政府决策、执行、监督的全过程，切实将行政权力纳入法制轨道，严格按照法定权限和程序行使权力、履行职责。依法接受人大及其常委会的法律监督和工作监督，主动接受人民政协的民主监督，自觉接受社会各界和人民群众的监督。认真办理人大代表建议和政协提案，做好人大执法检查、代表视察和政协委员调研成果的采纳和落实工作。完善科学民主决策机制，提高政府决策水平，健全政府督查机制。认真落实行政执法责任制和执法过错追究制，严格执法、文明行政。

清正廉洁，提高秉公用权的公信力。各级干部要严格执行廉洁从政的各项规定，珍重人格、珍爱声誉、珍惜形象，把廉洁当成一种职责要求、一种生活态度、一种精神追求，修身慎行，怀德自重，知足淡欲，清廉自守，永葆人民公仆本色。不断拓宽公共资源市场化配置领域，着力抓好对工程建设、房地产开发、土地管理和矿产资源开发等领域监督。加强监察、审计工作，防止权力失控、决策失误、行为失范。深入治理商业贿赂，严肃查处违纪违法案件，切实纠正损害群众利益的不正之风。着力建设科学严密、完备管用的反腐倡廉制度体系，提高廉政建设总体水平。

转变职能，提高公共服务的履行力。切实把政府职能转到创造良好环境、提供公共服务、维护公平正义上来。切实把经济发展、社会和谐、人民富裕作为各级政府的执政使命和追求，不断增强人民幸福感和社会和谐度。把更多注意力放到社会管理上，把更多精力投入到公共服务上，把更多财力倾斜到改善民生上。加强政风行风评议和机关效能建设，强化绩效管理。深化行政审批制度改革，充分发挥行政服务中心职能，推行网上审批和电子监察工作。继续做好政府信息公开工作。不断提高政府服务水平，积极主动地帮助企业，满腔热情地关心群众，为市场主体投资创业提供优质服务，为人民群众安居乐业创造良好环境。

各位代表，莆田正处在新一轮加快发展的关键时期，我们所面临的发展任务比任何时候更为繁重和紧迫。我们坚信，任何困难都改变不了兴化儿女创造幸福生活的强烈愿望，任何挑战都阻挡不住莆田人民加快发展的坚定步伐。让我们在中共莆田市委的坚强领导下，全面贯彻落实科学发展观，进一步解放思想，开拓进取，凝心聚力，乘势而上，为绘就湄洲湾港口城市繁荣富强新画卷、谱写莆田人民美好生活新篇章而努力奋斗！
